# Prevalence of liver cirrhosis in individuals with hepatitis B virus infection in sub‐Saharan Africa: Systematic review and meta‐analysis

**DOI:** 10.1111/liv.14744

**Published:** 2020-12-12

**Authors:** Bernard Surial, Dominik Wyser, Charles Béguelin, Adrià Ramírez‐Mena, Andri Rauch, Gilles Wandeler

**Affiliations:** ^1^ Department of Infectious Diseases Inselspital, Bern University Hospital, University of Bern Bern Switzerland; ^2^ Institute of Social and Preventive Medicine University of Bern Bern Switzerland

**Keywords:** antiviral treatment, cirrhosis, Hepatitis B virus, liver fibrosis, Sub‐Saharan Africa, systematic review

## Abstract

**Background & Aims:**

Chronic hepatitis B virus (HBV) infection accounts for 30%‐50% of cirrhosis related deaths in sub‐Saharan Africa (SSA). Since HBV‐related cirrhosis is an indication for immediate antiviral therapy and cancer surveillance, we aimed to estimate the prevalence of cirrhosis among treatment‐naïve patients with chronic HBV infection in SSA.

**Methods:**

We performed a systematic review of published articles which evaluated liver fibrosis stage among treatment‐naïve HBV‐infected individuals who presented to care in SSA. Our primary outcome was the prevalence of cirrhosis in HBsAg‐positive persons, which was estimated using random‐effects meta‐analysis. Risk factors for cirrhosis were explored using subgroup‐analyses and multivariable meta‐regression.

**Results:**

Of 2129 articles identified, 17 met our eligibility criteria. The studies described 22 cohorts from 13 countries, including 13 cohorts (3204 patients) with chronic HBV mono‐infection and nine cohorts (688 patients) with HIV/HBV‐coinfection. Liver fibrosis was assessed using transient elastography (10 cohorts), APRI score (11 cohorts), and Fibrotest (one cohort). The pooled prevalence of cirrhosis was 4.1% (95% confidence interval [CI] 2.6‐6.4) among studies from primary care facilities or general population, compared to 12.7% (95% CI 8.6‐18.3) in studies performed in referral or tertiary care facilities (adjusted odds ratio 0.29, 95% CI 0.15‐0.56). We found no association between cirrhosis and age, gender, fibrosis test used or HIV‐coinfection.

**Conclusions:**

Depending on the setting, between 4% and 13% of HBV‐infected individuals in SSA have cirrhosis and need immediate antiviral therapy. These estimates should be considered when planning HBV treatment strategies and resource allocation.

**Abbreviations**
APRI: AST‐to‐platelet ratio index
CENTRAL: Cochrane Central Register of Controlled Trials
CI: confidence interval
FIB‐4: fibrosis‐4
HBsAg: hepatitis B virus surface antigen
HBV: hepatitis B virus
HCC: hepatocellular carcinoma
PRISMA: Preferred Reporting Items for Systematic Review and Meta‐Analyses
PROSPERO: International Prospective Register of Systematic Reviews
SSA: sub‐Saharan Africa
TE: transient elastography
WHO: World Health Organization

**Key points**

- Liver cirrhosis is the most important driver of hepatitis B virus (HBV)‐related morbidity and mortality.
- Individuals with liver cirrhosis should receive immediate antiviral treatment with tenofovir and screening for hepatocellular carcinoma (HCC).
- In this meta‐analysis of 17 studies evaluating liver fibrosis stage among treatment‐naïve HBV‐infected individuals in sub‐Saharan Africa (SSA), the pooled prevalence of liver cirrhosis was 6.7% and differed significantly between primary care facilities (4.1%) and tertiary care or referral hospitals (12.7%).
- Based on our findings, an estimated 2.5 of 60 million individuals with chronic HBV infection in SSA have liver cirrhosis and need antiviral therapy and surveillance for liver cancer.

## 1 INTRODUCTION

Chronic hepatitis B virus (HBV) infection is the leading cause of liver‐related deaths worldwide and accounts for around one third of all deaths attributed to cirrhosis. In sub‐Saharan Africa (SSA), where most infections occur at birth or during early childhood, 30%‐50% of all cirrhosis‐related deaths can be attributed to chronic HBV infection.^1^ Antiviral treatment of patients with chronic HBV infection prevents the progression of cirrhosis^2^ and reduces the incidence of hepatocellular carcinoma (HCC) in patients with and without HIV infection,^3^, ^4^ thereby decreasing HBV‐related morbidity and mortality. Although treatment eligibility criteria for individuals without cirrhosis vary across guidelines and their interpretation is often subject to debate, the presence of cirrhosis is a clear indication for antiviral therapy and ultrasound‐based HCC screening.^5^, ^6^ Large studies of HBV‐monoinfected^4^, ^7^ and HIV/HBV‐coinfected individuals^3^ have confirmed the increased risk of developing HCC among individuals with cirrhosis.

However, few large‐scale studies have evaluated the prevalence of HBV‐related liver fibrosis and cirrhosis in SSA, where the presence of additional environmental and infectious risk factors may further accelerate the progression of liver disease. Thus, knowing the prevalence of HBV‐related cirrhosis is crucial for evaluating antiviral therapy needs for SSA. In 2016, the World Health Organization (WHO) started to advocate for the elimination of viral hepatitis as a public health problem. Specific objectives include the diagnosis of 90% of all individuals with chronic HBV infection, and the treatment of 80% of eligible individuals by 2030.^8^ In order to inform global resource allocation strategies, we performed a systematic review and meta‐analysis to assess the prevalence of cirrhosis among treatment‐naïve HBV‐infected individuals in SSA.

## 2 METHODS

### 2.1 Search strategy and selection criteria

We performed a systematic search of PubMed, EMBASE, African Index Medicus (via Global Index Medicus), Cochrane Central Register of Controlled Trials (CENTRAL), Web of Science and CINAHL to identify articles, which reported the proportion of treatment‐naïve HBsAg‐positive individuals who presented with cirrhosis (see appendix for the detailed search strategy). We considered published papers from inception until June 25, 2020 with original data in which liver fibrosis was assessed systematically in cohorts from sub‐Saharan Africa. We considered all types of liver fibrosis assessments, including liver biopsy, transient elastography and serological scores such as the AST‐to‐platelet ratio index (APRI), fibrosis‐4 (FIB‐4) and Fibrotest. Studies which selected participants based on symptoms or stage of liver disease, and studies which assessed less than 20 HBV‐infected individuals were excluded. We did not apply any restrictions on time frame, age of study participants or language of publication. Title, abstract and full text screening as well as data extraction were performed independently by four investigators (DW, BS, CB and GW) and discrepancies were discussed until a consensus was reached. Risk of bias was assessed using a critical appraisal tool for prevalence studies.^9^ The reporting of the study follows the Preferred Reporting Items for Systematic Review and Meta‐Analyses (PRISMA) guidelines. The protocol for this systematic review and meta‐analysis was registered with the International Prospective Register of Systematic Reviews (PROSPERO, registration number CRD42020180422).

### 2.2 Outcomes and definitions

The primary outcome was the proportion of treatment‐naïve HBV‐infected individuals with cirrhosis, whereas the prevalence of significant liver fibrosis was assessed as a secondary outcome. We used the transient elastography (TE) cut‐offs reported by the authors which varied between studies. One study^10^ used a transient elastography cut‐off of >9.3 kPa to define significant fibrosis; as this value is above the WHO‐recommended threshold for significant fibrosis in HBV infection (>7.5‐8.5 kPa),^5^ we re‐categorized individuals with a value >9.3 kPa into the cirrhosis group. In another study, a substantially lower than the WHO‐recommended threshold was used to define significant fibrosis (≥5.9 kPa)^11^; since the authors provided estimates based on transient elastography values ≥7.6 kPa, we used these numbers to define significant fibrosis. APRI scores above 1.5 were categorized as significant fibrosis, and above 2.0 as cirrhosis.^5^ Studies, which reported data from more than one country or more than one patient population were separated into cohorts. If more than one method of fibrosis assessment was available, we prioritized transient elastography, followed by APRI and FIB‐4. For each cohort, only one method of assessment was used to calculate the prevalence, and only the first fibrosis assessment (prior to any antiviral treatment) was considered when repeated measurements were available.

### 2.3 Data analysis

The prevalence of cirrhosis and significant fibrosis was estimated using random intercept logistic regression and logit transformed proportions. The 95% confidence intervals (CI) were derived using the exact method, and statistical heterogeneity was assessed using the Cochrane's *Q* and the *I* ^2^ statistic. To explore potential causes of heterogeneity, we performed subgroup analyses according to the test used (transient elastography, APRI, FIB‐4 and Fibrotest^®^), cohort category (referral/teaching hospital and primary care/general population), and whether HIV co‐infection was present or not. In addition, we performed sensitivity analyses including only studies which used TE, the test with highest sensitivity and specificity for detecting liver fibrosis and cirrhosis.^5^ A sensitivity analysis restricted to studies performed in HBV‐monoinfected populations was performed to assess the impact of cohort categories in this subpopulation. Risk factors for cirrhosis were modelled using univariable and multivariable meta‐regression. All analyses were performed using the *meta* ^12^ package for R version 4.0.

## 3 RESULTS

### 3.1 Description of the study population

**FIGURE 1:**
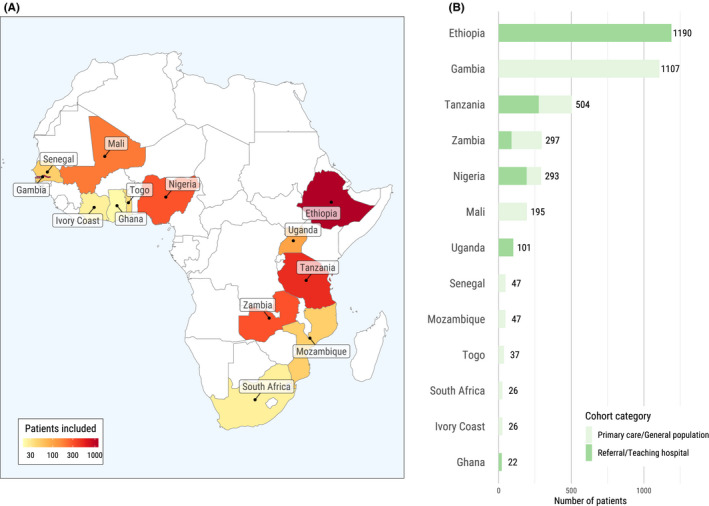
Overview of cohorts contributing data to the meta‐analysis. Countries with available data (A) and distribution of cohorts with data from primary care/general population cohorts or referral/teaching hospitals (B)

Of 2129 potential articles obtained through our systematic search, 17 met our eligibility criteria (Figure S1). The studies described 22 different cohorts including 3892 patients with chronic HBV infection from 13 different countries in sub‐Saharan Africa: Ethiopia (1), the Gambia (3), Ghana (1), Ivory Coast (1), Mali (1), Mozambique (1), Nigeria (3), Senegal (1), South Africa (1), Tanzania (4), Togo (1), Uganda (1) and Zambia (2, Figure 1). Fourteen cohorts included patients from the general population (eg primary care or screening of blood donors, 2021 patients [51.9%]), and eight studies recruited patients from referral centers or teaching hospitals (1871 patients [48.1%]). The largest contributions to the study populations stemmed from three cohorts from the Gambia (general population screening, n = 1107, 28.4%) and one cohort from Ethiopia (referral centre, n = 1190, 30.6%). Thirteen cohorts included HBV mono‐infected individuals (3204 patients, 82.3%), and nine cohorts included HIV/HBV coinfected individuals (688, 17.7%). The median age of the study population ranged from 27 to 38 years, and the proportion of females ranged from 0% to 65% (Table 1). Seven studies included information about the prevalence of HBeAg positivity, which ranged from 3% to 28%.

**TABLE 1:** Description of the cohorts included in the review

First author	Year	Country	Area	Cohort population	Cohort category	Study type	Individuals with HBV infection	Median age (years)	Women (%)	Tests used
HBV mono‐infected individuals										
Aberra H^13^	2019	Ethiopia	urban	Outpatient clinic	Referral/teaching hospital	prospective cohort	1190	31	43%	TE, APRI
Hawkins C^14^	2017	Tanzania	urban	Outpatient clinic	Primary care/general population	cross‐sectional	165	32	33%	APRI, FIB‐4
Iroezindu MO^15^	2013	Nigeria	urban	Outpatient clinic	Primary care/general population	cross‐sectional	100	34	61%	APRI, FIB‐4
Jaquet A^16^	2017	Togo	urban	Prisoners	Primary care/general population	cross‐sectional	37	30	0%	TE
Jaquet A^16^	2017	Senegal	urban	Prisoners	Primary care/general population	cross‐sectional	47	31	0%	TE
Jaquet A^16^	2017	Ivory Coast	urban	Outpatient clinic	Primary care/general population	cross‐sectional	26	29	54%	TE
Laing N^17^	2019	Uganda	urban	Outpatient clinic	Referral/teaching hospital	prospective cohort	101	27	53%	APRI
Lemoine M^18^	2016	Gambia	urban	Blood donors	Primary care/general population	cross‐sectional	300	31	3%	TE
Lemoine M^18^	2016	Gambia	both	Community screening	Primary care/general population	cross‐sectional	402	38	52%	TE
Shimakawa Y^19^	2016	Gambia	rural	Outpatient clinic	Primary care/general population	prospective cohort	405	n.a.	50%	TE
Traoré F^20^	2015	Mali	urban	Occupational group	Primary care/general population	cross‐sectional	195	35	27%	Fibrotest
Vinikoor MJ^21^	2018	Zambia	urban	Outpatient clinic	Referral/teaching hospital	cross‐sectional	89	33	39%	TE, APRI
Vinikoor MJ^22^	2020	Zambia	both	Community screening	Primary care/general population	Cross‐sectional	147	31	52%	APRI
HIV/HBV co‐infected individuals										
Anabire NG^23^	2019	Ghana	urban	Outpatient clinic	Referral/teaching hospital	cross‐sectional	22	33	64%	APRI
Bell TG^24^	2012	South Africa	rural	Outpatient clinic	Primary care/general population	cross‐sectional	26	34	50%	APRI
Chambal LM^25^	2017	Mozambique	urban	Outpatient clinic	Primary care/general population	cross‐sectional	47	32	64%	FIB‐4, APRI
Hawkins C^10^	2013	Nigeria	urban	Outpatient clinic	Referral/teaching hospital	cross‐sectional	93	33	n.a.	TE
Hawkins C^14^	2017	Tanzania	urban	Outpatient clinic	Primary care/general population	cross‐sectional	63	37	52%	APRI, FIB‐4
Iroezindu MO^15^	2013	Nigeria	urban	Outpatient clinic	Referral/teaching hospital	cross‐sectional	100	34	61%	APRI, FIB‐4
Kilonzo SB^26^	2017	Tanzania	urban	Outpatient clinic	Referral/teaching hospital	cross‐sectional	49	n.a.	65%	APRI, FIB‐4
Ramirez‐Mena A^27^	2016	Tanzania	rural	Outpatient clinic	Referral/teaching hospital	prospective cohort	227	37	52%	APRI
Vinikoor MJ^11^	2017	Zambia	urban	Outpatient clinic	Primary care/general population	prospective cohort	61	35	46%	TE

Note

APRI, ASAT‐to‐platelet ratio index; FIB‐4 fibrosis‐4; TE, transient elastography.

The assessment of liver fibrosis or cirrhosis relied mostly on TE (10 cohorts), followed by APRI and FIB‐4 (11 cohorts), and Fibrotest® (one cohort). No study reported results from liver biopsy. Studies used TE cut‐off values ranging from 9.4 to 12.3 kPa for defining cirrhosis and from 5.9 to 9.3 kPa for significant fibrosis. Estimates for liver fibrosis were reported in all but three cohorts, and cirrhosis was reported in 16 cohorts.

### 3.2 Prevalence of liver cirrhosis

**FIGURE 2:**
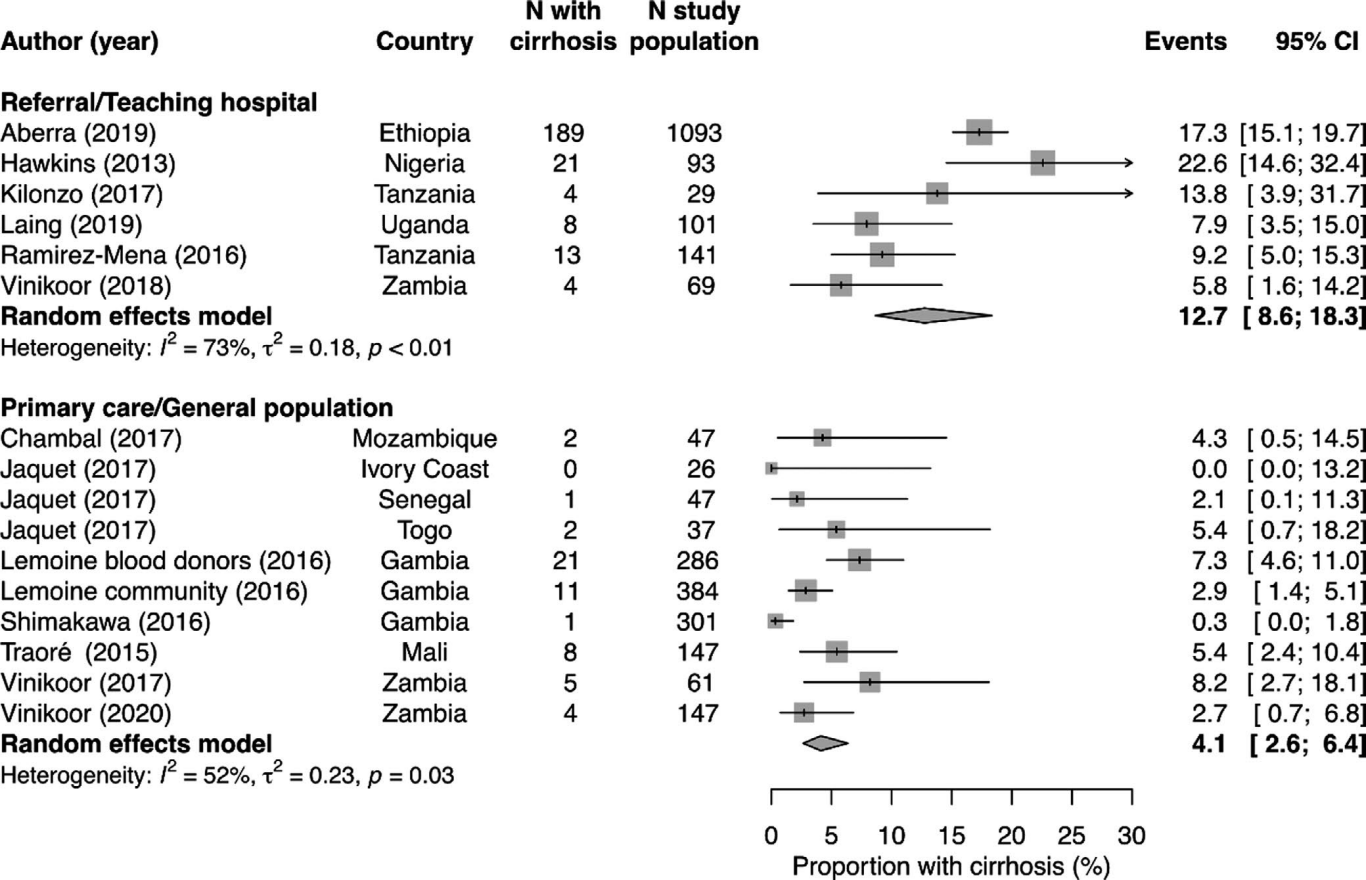
Proportion of individuals with liver cirrhosis, stratified by cohort category

**FIGURE 3:**
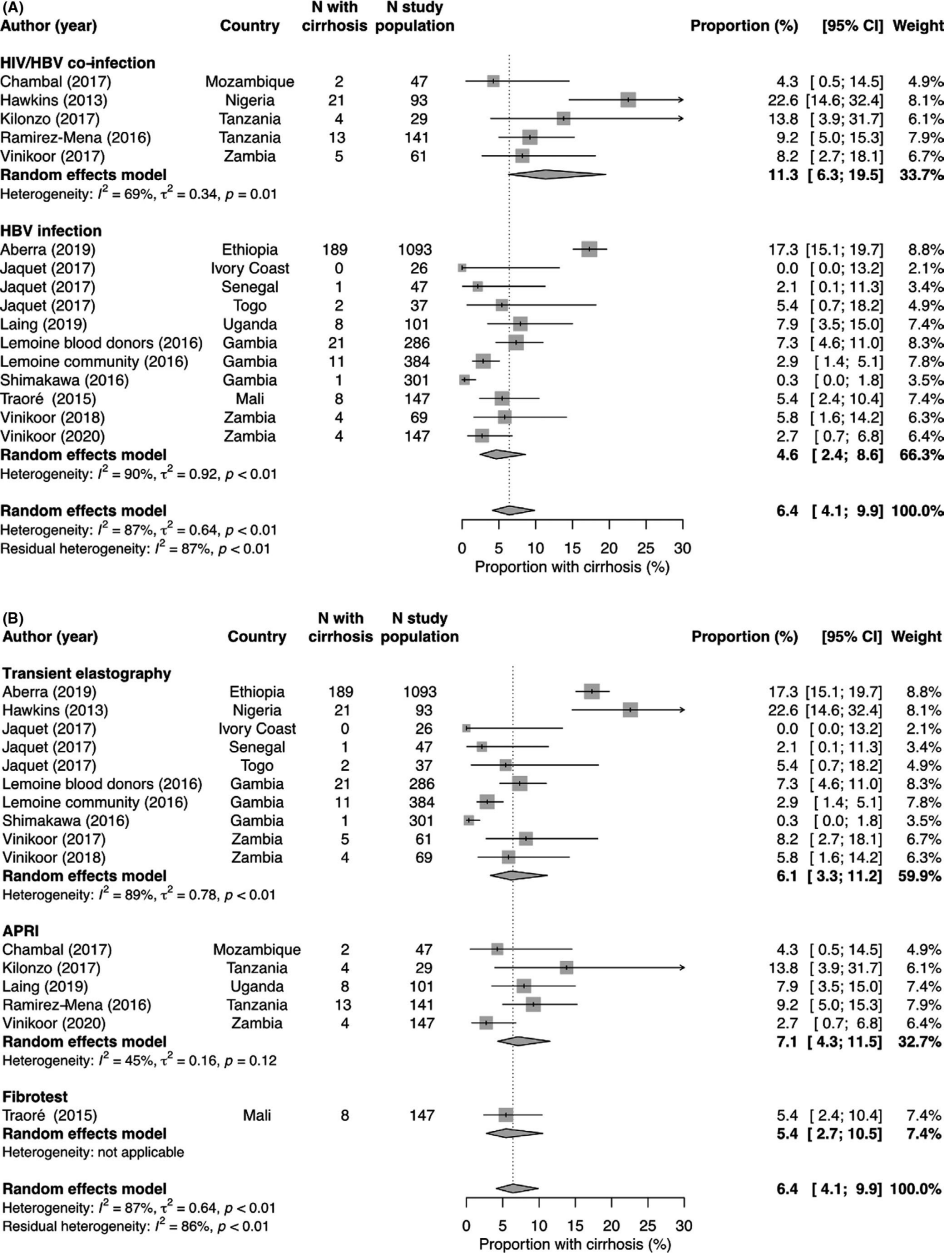
Proportion of individuals with liver cirrhosis, stratified by HIV infection status (A) and by fibrosis test used(B).

The pooled prevalence of cirrhosis was 6.4% (95% confidence interval [CI] 4.1‐9.9, *I* ^2^ = 87%) overall, with estimates from individual studies varying between 0% in a small cohort of HBV‐monoinfected individuals in Ivory Coast^16^ and 22.6% among HIV/HBV‐coinfected individuals in Nigeria.^10^ The most important factor contributing to heterogeneity of cirrhosis prevalence was the setting in which the study was performed: Whereas the prevalence was 12.7% (95% CI 8.6‐18.3, *I* ^2^ = 73%) in studies performed at referral or teaching hospitals, the estimate from studies performed in primary care facilities or as part of general population screening was 4.1% (95% CI 2.6‐6.4, *I* ^2^ = 52%, *P*‐value for between‐group difference <0.001, Figure 2). The pooled prevalence of cirrhosis was higher among HIV/HBV co‐infected individuals (11.3%, 95% CI 6.3‐19.5, *I* ^2^ = 69%) than in individuals without HIV infection (4.6%, 95% CI 2.4‐8.6, *I* ^2^ = 90%, *P*‐value for between‐group difference 0.04, Figure 3A). Studies based on transient elastography reported a cirrhosis prevalence of 6.1% (95% CI 3.3‐11.2, *I* ^2^ = 89%), compared to 7.1% in studies based on APRI, (95% CI 4.3%‐11.5, *I* ^2^ = 45%), and 5.4% (95% CI 2.7%‐10.5%) in the one which used Fibrotest (*P* for between‐group difference 0.81, Figure 3B). Neither percentage of women within the studies (*P* = .90), median age of the study population (*P* = .78), fibrosis test used (*P* = .87), nor HIV coinfection status (*P* = .10) were significantly associated with the prevalence of cirrhosis in univariable meta‐regression (Table S1). In multivariable meta‐regression adjusted for co‐infection status and test used, study setting was the only factor which remained significantly associated with cirrhosis (adjusted odds ratio 0.29 for cohorts from primary care or general population screening vs referral or teaching hospitals, 95% CI 0.15‐0.56).

A sensitivity analysis including only studies which used transient elastography to evaluate cirrhosis revealed similar results: The overall pooled prevalence of cirrhosis was 6.1% (95% CI 3.3‐11.2, *I* ^2^ = 89%), whereas the estimate was 15.9% (95% CI 10.0‐24.4, *I* ^2^ = 73%) in studies at referral or teaching hospitals, and 3.8% (95% CI 2.0‐7.3, *I* ^2^ = 65%) among studies performed in primary care or as general population screening (residual heterogeneity *I* ^2^ *= *67%, Figure S2 *)*. After restricting the main analysis to studies performed in HBV‐monoinfected individuals, studies at referral or teaching hospitals remained associated with a higher prevalence of cirrhosis (10.3%, 95% CI 4.8‐20.7, *I* ^2^ = 81%) compared to studies from primary care or general population cohorts (3.6%, 95% CI 2.1‐6.1, *I* ^2^ = 59%, Figure S3). To further examine the robustness of our results, we excluded the largest study from Aberra et al (n = 1190) in an additional analysis, and found similar estimates (Figure S4).

### 3.3 Prevalence of significant liver fibrosis

The proportion of individuals with significant fibrosis ranged from 4.0% to 35.4% across cohorts, with an overall pooled prevalence of 12.0% (95% CI 8.8‐16.4, *I* ^2^ = 88%). There was no significant difference between cohorts from primary care or general populations (10.4%, 95% CI 6.4‐16.8, *I* ^2^ = 90%) and those from referral or teaching hospitals (16.4%, 95% CI 11.9‐22.3, *I* ^2^ = 64%), and between‐study heterogeneity was high (Figure S5). The pooled prevalence was similar among studies which used transient elastography (11.6%, 95% CI 7.4‐17.8, *I* ^2^ = 91%) and those which used APRI (11.2%, 95% CI 7.2%‐17.0%, *I* ^2^ = 64%), whereas the prevalence was lower in the cohort which relied on FIB‐4 (4.3%, 95% CI 1.1%‐15.5%), and higher in the cohort which used Fibrotest® (35.4%, 95% CI 28.1%‐43.4%).

### 3.4 Assessment of study quality

Most studies were performed among a sample of individuals representative of the target population of the specific study, and all studies measured the outcome in a standardized and reliable way within their study population. Whereas most studies reported a systematic screening of HIV among all participants, one study did not state whether HIV was screened for.^20^ However, some studies failed to describe how patients were selected into the studies, and only a minority of studies performed random sampling of eligible patients. In addition, the sample size was small in most studies, and the main outcome was incompletely assessed in three studies. Whereas 50% of all studies relied on transient elastography to assess liver fibrosis, the less sensitive APRI method was mainly used in studies of HIV/HBV co‐infected patients (Table S2).

## 4 DISCUSSION

Among treatment‐naïve HBV‐infected individuals in sub‐Saharan Africa, the prevalence of cirrhosis was 4.1% (95% CI 2.6‐6.4) in studies performed at primary care facilities or as part of general population screening, compared to 12.7% (95% CI 8.6‐18.3) in studies performed at referral or teaching hospitals. Substantial heterogeneity was observed between studies, and the majority of them reported outcomes from cohorts of less than 200 patients. Nevertheless, our study provides a basis for estimating the number of HBV‐infected individuals in need of immediate antiviral therapy because of the presence of cirrhosis in sub‐Saharan Africa.

The type of study setting had a strong impact on the prevalence of cirrhosis observed: Studies from tertiary care centres reported a prevalence of up to 22%, which arguably reflected the fact that patients with more advanced diseases were referred to such centres for specialized care. The estimate of 4.1% derived from studies from primary care and population screening was fairly robust and is more likely to represent the prevalence in the general population in sub‐Saharan Africa. The difference in prevalence between the two types of setting is illustrated by the comparison of the estimates of the two largest studies: Among 402 individuals identified through community‐based screening in the Gambia, the prevalence of cirrhosis was 2.8%^18^; in contrast, of 1190 individuals with chronic HBV infection referred to a teaching hospital in Ethiopia, 17.3% were diagnosed with cirrhosis.^13^

The studies included varied in terms of the methods used to assess liver fibrosis and cirrhosis. Whereas there was substantial heterogeneity among studies reporting on cirrhosis, we found no evidence that the method of cirrhosis assessment contributed significantly to the estimates. This finding was surprising, since the sensitivity of APRI to diagnose cirrhosis is considered low, leading to many cases of cirrhosis being missed.^5^ Studies from Europe and Ethiopia have shown that between two‐thirds and 80% of individuals with HBV‐related cirrhosis are missed when fibrosis is assessed with the APRI score.^13^, ^28^ However, since we were unable to compare transient elastography and APRI scores within the same studies, and only few large studies using APRI were available for this review, the analyses on the impact of fibrosis assessment method on cirrhosis prevalence should be interpreted with caution.

HIV co‐infection can significantly alter the course of HBV‐related liver disease progression. Whereas co‐infected patients seem to be more likely to develop chronic infection, it is unclear whether and to what extent HIV/HBV co‐infection influences the development of cirrhosis in sub‐Saharan Africa, where HBV infection is generally acquired years before HIV infection.^29^ In our meta‐analysis, studies performed in HIV/HBV co‐infected patients reported a higher prevalence of cirrhosis compared to studies in HBV mono‐infected patients, but co‐infection was not significantly associated with cirrhosis in multivariable meta‐regression. Comparative studies between HBV mono‐infected and HIV/HBV‐coinfected individuals within the same settings are needed to better understand the potential impact of HIV infection on HBV‐related cirrhosis.

Our study provides robust pooled estimates of the prevalence of cirrhosis among HBV‐infected individuals in sub‐Saharan Africa, which will help determine and plan HBV treatment needs. Meta‐regression analyses allowed us to confirm the strong association between study setting and cirrhosis prevalence, whereas the role of HIV infection seemed less evident. However, the substantial residual heterogeneity of the subgroup analysis stratified by study setting indicates that other explanatory factors might not have been captured in our study. For instance, we relied on the cut‐offs for transient elastography defined in each individual study, albeit the thresholds used in the studies differed significantly, potentially contributing to between‐study heterogeneity. This was especially true for studies performed among HIV/HBV co‐infected individuals, a population for which optimal transient elastography cut‐offs remain ill‐defined. Despite a broad literature search without language and date restrictions, we found only few large studies, which assessed the presence of cirrhosis with transient elastography, and the low number of studies limited the statistical power to reliably detect differences between subgroups. Close to two‐thirds of the individuals included in our meta‐analysis were from cohorts in the Gambia and Ethiopia, and many African countries were not represented in our analyses. Finally, there was only limited information about the proportion of patients with a positive HBeAg in most studies. Differences in the prevalence of HBeAg‐positivity may have had an impact on the progression of liver fibrosis, as shown in studies from other regions.^30^

In summary, we show that a significant proportion of the HBV‐infected population in sub‐Saharan Africa has cirrhosis. Our findings have important policy implications for resource allocation strategies and national HBV treatment programs in order to achieve the WHO elimination goals by 2030. According to the WHO, only 150 000 HBV‐infected individuals had been diagnosed by 2015 in sub‐Saharan Africa, and 18% of them had received antiviral treatment.^8^ Based on the results from our meta‐analysis, 2.5 Million of the estimated 60 Million people with chronic HBV infection in sub‐Saharan Africa have cirrhosis and need immediate antiviral therapy and HCC screening.^8^, ^31^ More efforts are needed to identify HBV‐infected individuals in SSA, and to systematically assess the extent of HBV‐associated liver disease. Since widely available tests such as the APRI score miss a significant proportion of cases of cirrhosis, there is an urgent need for access to more accurate cirrhosis assessment tools such as transient elastography in order to identify patients for whom HBV therapy and surveillance are crucial.

## CONFLICT OF INTEREST

BS reports support to his institution for travel grants from Gilead Sciences. AR reports support to his institution for advisory boards and/or travel grants from Janssen‐Cilag, MSD, Gilead Sciences, and Abbvie, and an unrestricted research grant from Gilead Sciences. All remuneration went to his home institution and not to AR personally, and all remuneration was provided outside the submitted work. GW reports support to his home institution for advisory boards and/or travel grants from MSD, Gilead Sciences and Abbvie, and an unrestricted research grant from Gilead Sciences. DW, CB and ARM report no conflicts of interest.

## AUTHOR’S CONTRIBUTION

CB and GW designed the study. DW, BS, CB and GW performed the literature search, study selection and data extraction. BS performed the statistical analyses. BS, DW and GW wrote the first draft of the manuscript. All authors contributed to the interpretation of the data and critically reviewed the manuscript.

## Funding information

GW is supported by a Professorship from the Swiss National Science Foundation (PP00P3_176944). The funders had no role in study design, data collection and analysis, decision to publish, or preparation of the manuscript. The corresponding author had full access to all the data in the study and had the final responsibility for the decision to submit for publication.

## Supporting information

Supplementary Material
