# Supplementary material for: Prevalence of liver cirrhosis in individuals with hepatitis B virus infection in sub‐Saharan Africa: Systematic review and meta‐analysis
Source: Liver Int. 2020 Dec 12;41(4):710–9. doi: 10.1111/liv.14744 (PMC8048614; doi:10.1111/liv.14744)
Supplement: Supplementary file 1 — Supplementary Material [file LIV-41-710-s001.docx]

## Supporting Information

### Search strategy for PubMed

((exp Hepatitis B OR (hepatitis B or hep B or HBV or CHB)) AND (exp Liver Cirrhosis OR ((hepatic or liver) and (fibros* or cirrho*)) OR (liver adj3 stiffness*))) AND (exp Africa OR (Africa*1 or Algeria or Angola or Benin or Botswana or Burkina Faso or Burundi or Cabo Verde or Cameroon or Central African Republic or CAR or Chad or Comoros or Democratic Republic of the Congo or Republic of the Congo or Cote d'Ivoire or Djibouti or Egypt or Equatorial Guinea or Eritrea or Ethiopia or Gabon or Gambia or Ghana or Guinea or Guinea-Bissau or Kenya or Lesotho or Liberia or Libya or Madagascar or Malawi or Mali or Mauritania or Mauritius or Morocco or Mozambique or Namibia or Niger or Nigeria or Rwanda or "Sao Tome and Principe" or Senegal or Seychelles or Sierra Leone or Somalia or South Africa or sub-Sahara* or Subsahara* or South Sudan or Sudan or Swaziland or Tanzania or Togo or Tunisia or Uganda or Zambia or Zimbabwe)).

#### Figure S1: Study selection


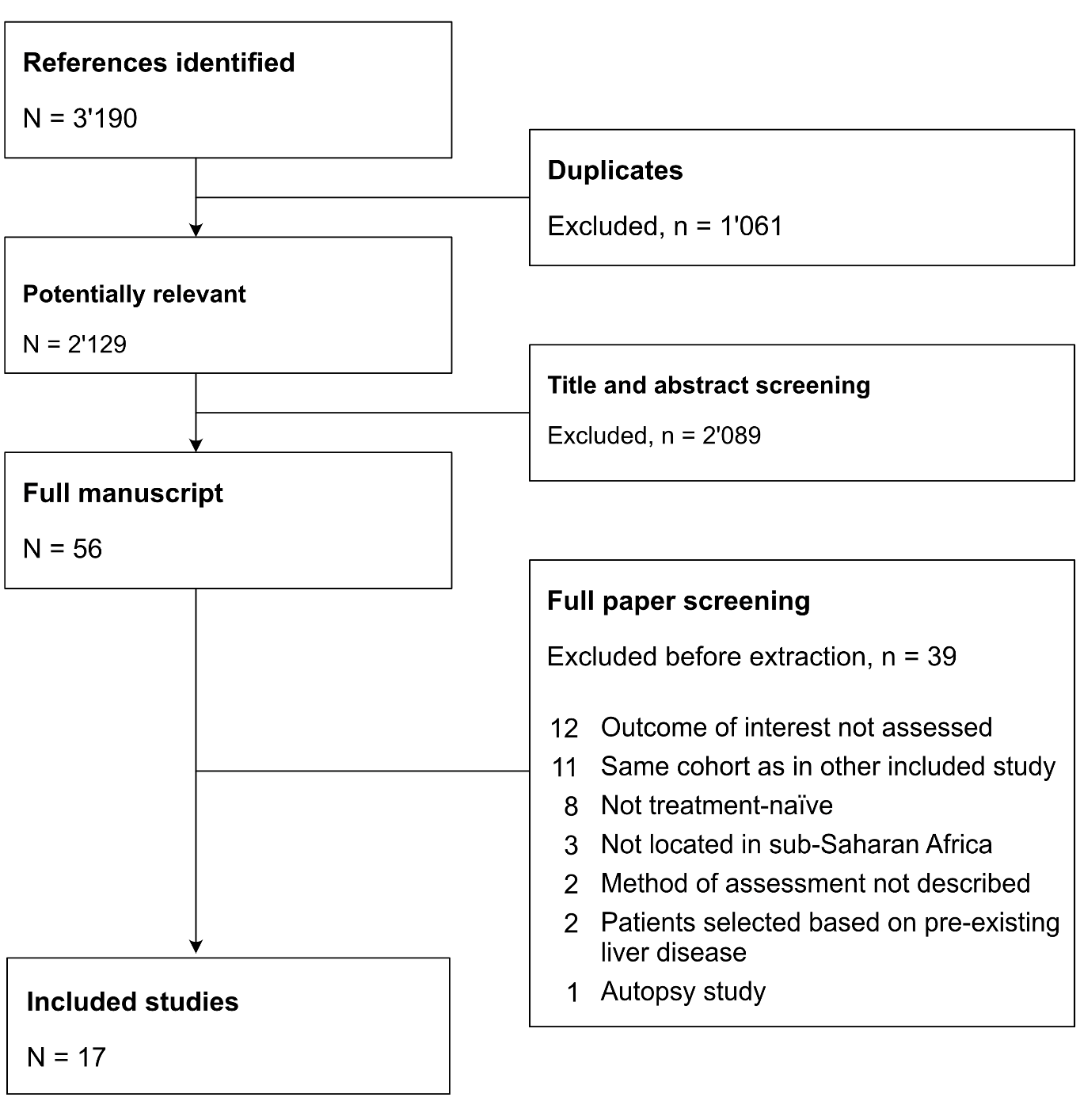


#### Figure S2: Proportion of individuals with liver cirrhosis, stratified by type of study population (restricted to studies which used transient elastography)


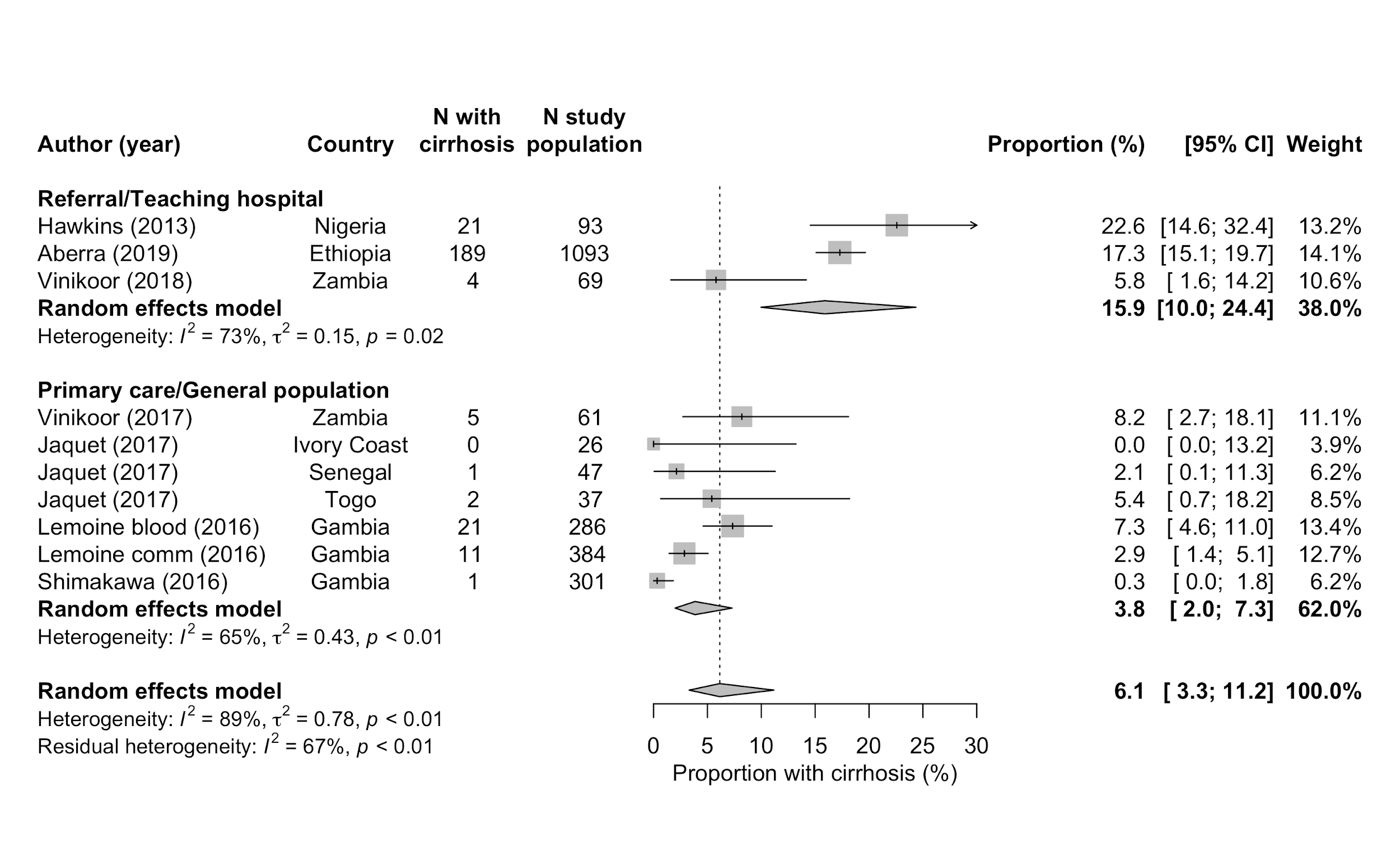


#### Figure S3: Proportion of individuals with liver cirrhosis, stratified by type of study population (restricted to studies among HBV-monoinfected individuals)


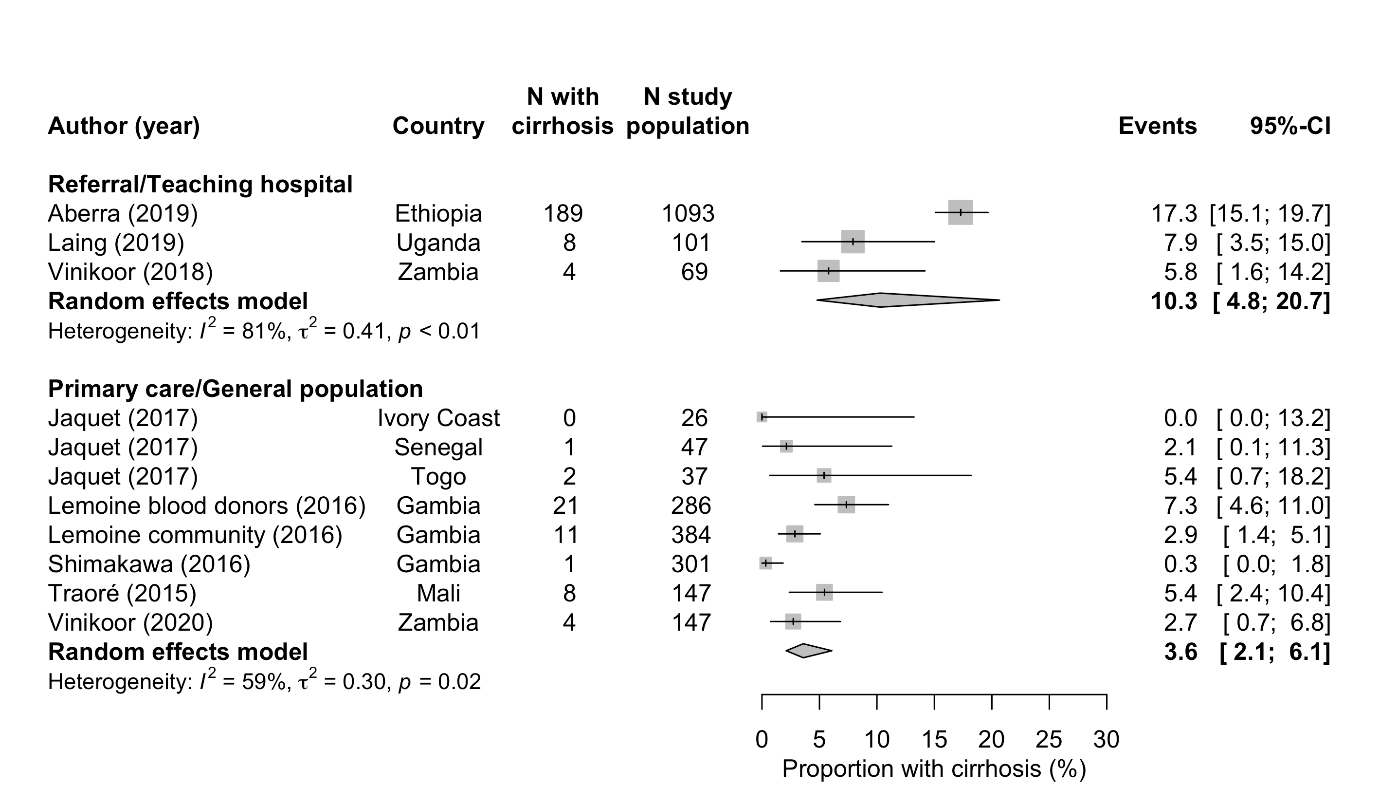


#### Figure S4: Proportion of individuals with liver cirrhosis, stratified by type of study population (after excluding the largest study from Aberra et al, 2019)


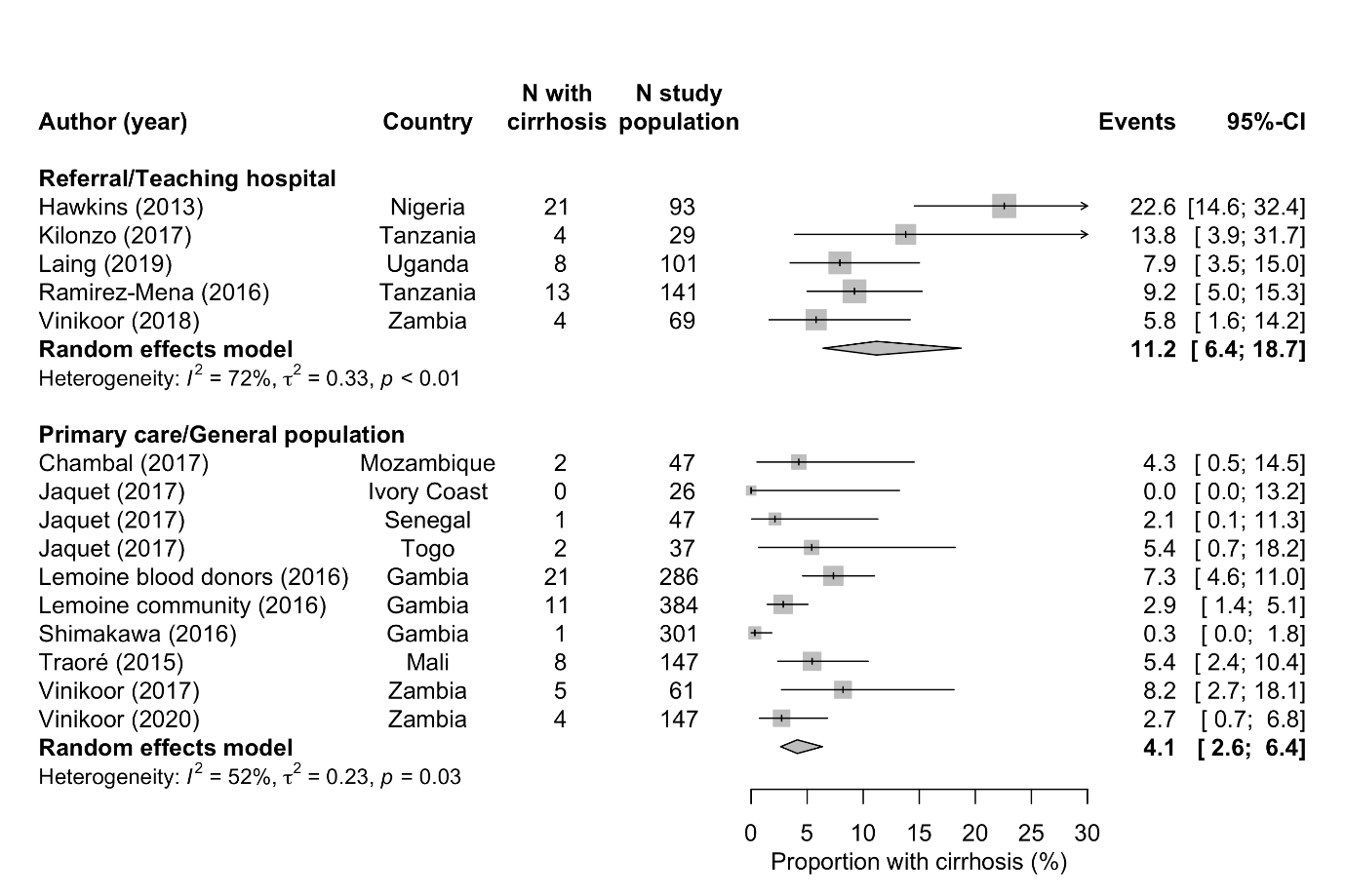


#### Figure S5: Proportion of individuals with significant liver fibrosis, stratified by type of study population


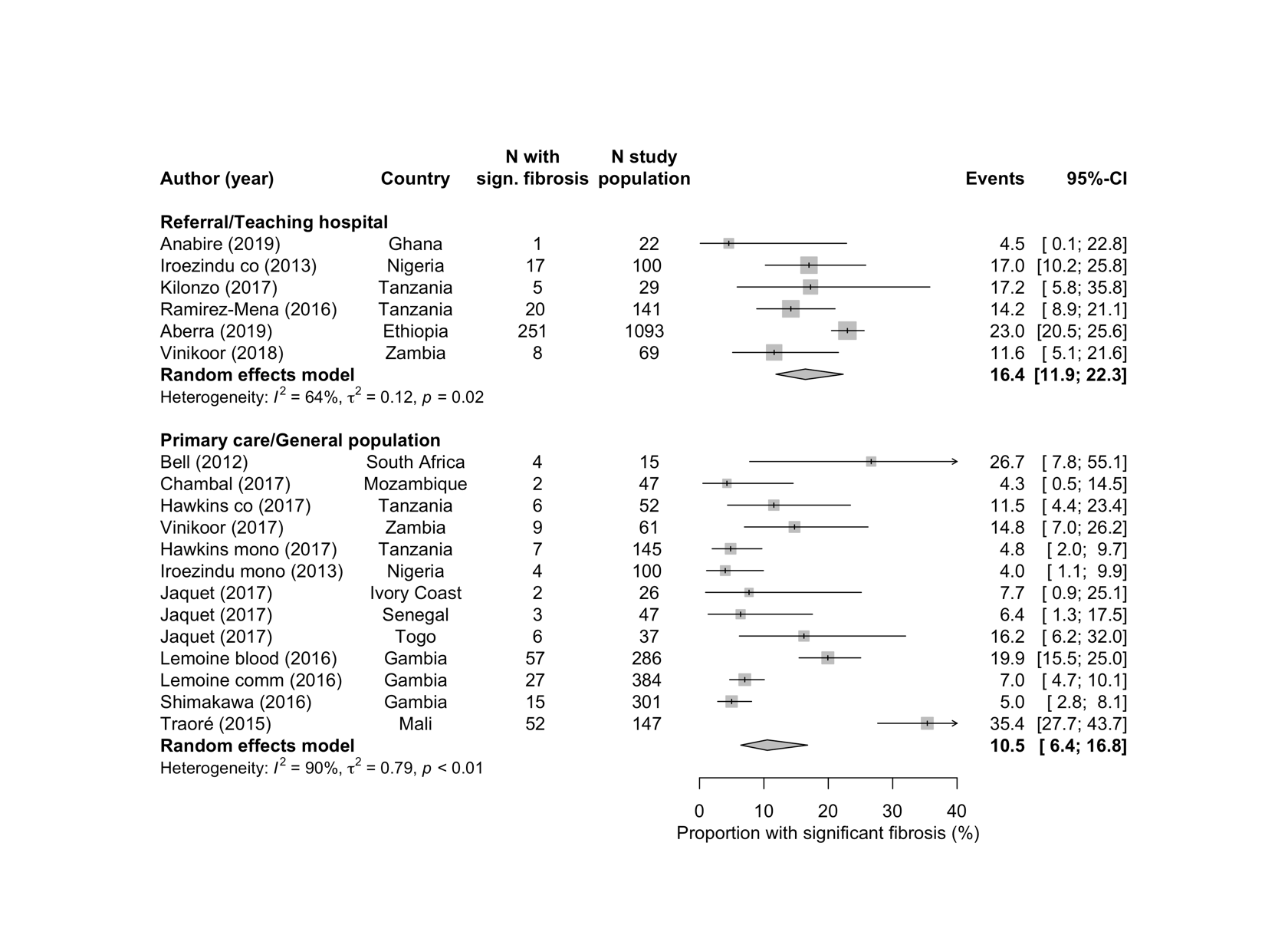


#### Table S1: Meta-regression for the prevalence of liver cirrhosis

| **Variable** |  | **Univariable regression** | |  | **Multivariable regression** | |
| --- | --- | --- | --- | --- | --- | --- |
|  |  | **univariable OR** | **p-value** |  | **adjusted OR** | **p-value** |
| **Proportion of female (per one % increase)** |  | 1·18 (0·09 to 15·71) | 0·90 |  | – |  |
| **Median age of study population (per one year increase)** |  | 0·98 (0·84 to 1·14) | 0·78 |  | – |  |
| **Cirrhosis test used** |  |  |  |  |  |  |
| Fibroscan |  | 1 |  |  | 1 |  |
| APRI |  | 1·09 (0·39 to 3·07) | 0·87 |  | 0·54 (0·26 to 1·09) | 0·09 |
| Fibrotest |  | 0·86 (0·13 to 5·50) | 0·87 |  | 1·38 (0·42 to 4·55) | 0·60 |
| **Cohort Setting** |  |  |  |  |  |  |
| Tertiary Care |  | 1 |  |  | 1 |  |
| Primary care / General population screening |  | 0·30 (0·16 to 0·56) | <0·001 |  | 0·29 (0·15 to 0·56) | <0·001 |
| **Infection status** |  |  |  |  |  |  |
| HIV/HBV coinfected |  | 1 |  |  | 1 |  |
| HBV mono-infected |  | 0·41 (0·14 to 1·20) | 0·10 |  | 0·55 (0·27 to 1·10) | 0·09 |
| **APRI** ASAT to platelet ratio index, **OR** odds ratio, **HBV** hepatitis B virus. | | | | | | |

### Table S2 Risk of bias assessment of included studies

|  | Sample representative of target population | Study participants recruited appropriately? | | Sample size adequate* | | Participants and setting described in detail | | Data analyzed with sufficient coverage of identified sample | | Valid methods used to identify the condition | | Condition measured in a standard, reliable way for all participants | | Appropriate statistical analysis | |
| --- | --- | --- | --- | --- | --- | --- | --- | --- | --- | --- | --- | --- | --- | --- | --- |
| **Study** |  |  | |  | |  | |  | |  | |  | |  | |
| Aberra (2019)^13^ |  |  | |  | |  | |  | |  | |  | |  | |
| Anabire (2019)^23^ |  |  | |  | |  | |  | |  | |  | |  | |
| Bell (2012)^24^ |  |  | |  | |  | |  | |  | |  | |  | |
| Chambal (2017)^25^ |  |  | |  | |  | |  | |  | |  | |  | |
| Hawkins (2013)^10^ |  |  | |  | |  | |  | |  | |  | |  | |
| Hawkins (2017)^14^ |  |  | |  | |  | |  | |  | |  | |  | |
| Iroezindu (2017)^15^ |  |  | |  | |  | |  | |  | |  | |  | |
| Jaquet (2017)^16^ |  |  | |  | |  | |  | |  | |  | |  | |
| Kilonzo (2017)^26^ |  |  | |  | |  | |  | |  | |  | |  | |
| Laing (2019)^17^ |  |  | |  | |  | |  | |  | |  | |  | |
| Lemoine (2016)^18^ |  |  | |  | |  | |  | |  | |  | |  | |
| Ramirez-Mena (2016)^27^ |  |  | |  | |  | |  | |  | |  | |  | |
| Shimakawa (2016)^19^ |  |  | |  | |  | |  | |  | |  | |  | |
| Traoré (2015)^20^ |  |  | |  | |  | |  | |  | |  | |  | |
| Vinikoor (2017)^11^ |  |  | |  | |  | |  | |  | |  | |  | |
| Vinikoor (2018)^21^ |  |  | |  | |  | |  | |  | |  | |  | |
| Vinikoor (2020)^22^ |  |  | |  | |  | |  | |  | |  | |  | |
|  |  | |  | |  | |  | |  | |  | |  | |  |
| **Legend** | Yes | | Unclear | | No | |  | |  | |  | |  | |  |
| * A minimum sample size of 140 was derived using the formula of Munn et al for an expected estimate of 10% and a precision of +/- 5%. | | | | | | | | | | | | | | | |
